# Intestinal and fecal pH in human health

**DOI:** 10.3389/frmbi.2023.1192316

**Published:** 2023-07-26

**Authors:** Ryodai Yamamura, Kumi Y. Inoue, Kunihiko Nishino, Seiji Yamasaki

**Affiliations:** ^1^ Institute for Genetic Medicine, Hokkaido University, Sapporo, Japan; ^2^ Graduate Faculty of Interdisciplinary Research, University of Yamanashi, Kofu, Japan; ^3^ Graduate School of Environmental Studies, Tohoku University, Sendai, Japan; ^4^ SANKEN (The Institute of Scientific and Industrial Research), Osaka University, Ibaraki, Japan; ^5^ Graduate School of Pharmaceutical Sciences, Osaka University, Suita, Japan; ^6^ Center for Infectious Disease Education and Research, Osaka University, Suita, Japan; ^7^ Institute for Advanced Co-Creation Studies, Osaka University, Suita, Japan

**Keywords:** gut microbiota, fecal pH, biomarkers, short-chain fatty acids, metabolites, metabolism, health, disease

## Abstract

Gut microbiota has been reported to be closely related to host energy metabolism and immunity, and thus influence the development and progression of various human diseases. To date, the gut microbial metabolites such as short-chain fatty acids, defensins, cathelicidins, and lactoferrin in feces have been investigated as biomarkers associated with various disease conditions. In this review, we introduce intestinal and fecal pH, which is relatively easy and rapid to measure compared to the composition of the gut microbiota and its metabolites. In particular, this review presents the distribution of pH in the human body, its role and clinical significance, and various factors that affect intestinal and fecal pH, including the gut microbiota and its metabolites.

## Introduction

In recent years, with dramatic advances in next-generation sequencers (NGS), many studies have revealed a link between the intestinal microbiota and the onset and progression of various human diseases. Diseases affected by the gut microbiota include not only gastrointestinal diseases such as gastrointestinal cancer and inflammatory bowel disease, but also metabolic diseases, cardiovascular diseases, neurodegenerative diseases, and even psychiatric disorders (Chen et al., 2021; Yamamura et al., 2021). In addition, high-throughput multi-omics analysis such as metabolomics has revealed that not only bacteria but also metabolites of the gut microbiota such as cathelicidin, lactoferrin, osteoprotegerin, S100 protein, M2-PK, and short-chain fatty acids (SCFAs) are correlated and altered with human health and diseases (Pang et al., 2014). Therefore, monitoring the intra-individual variability of these metabolites is expected to be useful in understanding health or disease conditions.

In addition to these microbiota and metabolites, fecal pH, which can be measured more easily, non-invasively, and rapidly, has been reported to be strongly associated with human health and has attracted much attention. Furthermore, although more invasive and more time consuming to measure than fecal pH, intestinal pH has made significant contributions, particularly in the development of systems to deliver drugs to specific locations (vander Schaar et al., 2013). For example, the enteric coating of 5-aminosalicylic acid (5-ASA), recommended as a first-line drug for patients with mild to moderate ulcerative colitis (UC), is designed to resist the highly acidic environment of the stomach and to dissolve in the basic environment (around pH 7) of the terminal ileum (Nugent et al., 2001).

Fecal pH can be rapidly measured using a commonly calibrated pH probe and meter (Cox et al., 2020; Gill et al., 2022), and intestinal pH can be measured with high precision in real time using telemetry capsules or oral tube-attached pH electrodes (Evans et al., 1988; Nugent et al., 2001; vander Schaar et al., 2013). In fact, fecal pH has traditionally been used clinically as an indicator of colonic carbohydrate malabsorption and osmotic diarrhea (see “Clinical Significance of Fecal pH” section), however, its importance remains questionable due to its large intra-individual as well as inter-individual variability. In this context, this review first describes the distribution and role of pH in the gastrointestinal tract and its role, then reviews the latest findings on the influences of various factors such as diet, disease, and drugs on intestinal and fecal pH variation in conjunction with the gut microbiota and its metabolites.

## pH distribution in the stomach and its roles

**Figure 1:**
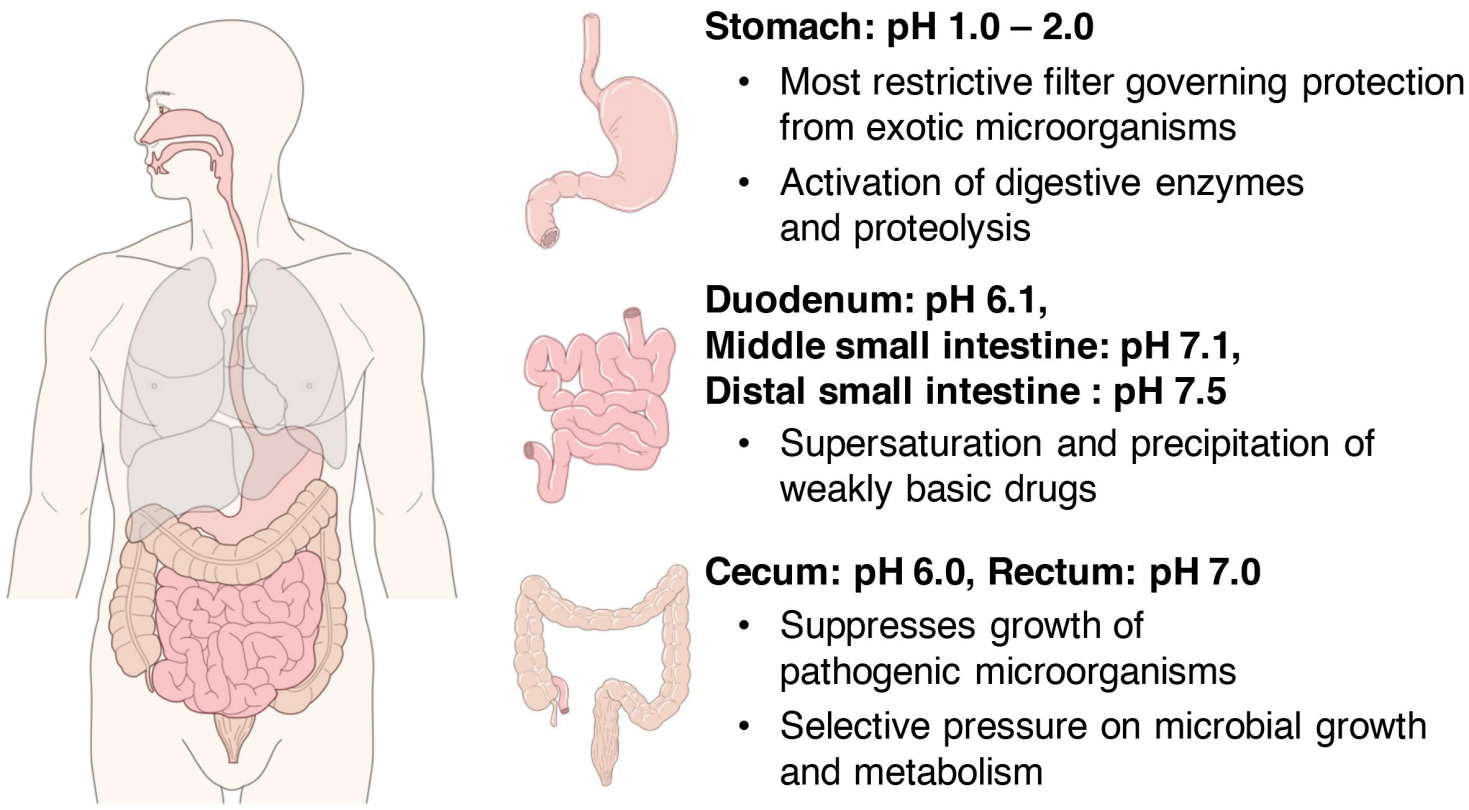
Distribution of pH in the gastrointestinal tract. In the stomach, strong bactericidal action is necessary, and gastric juices have a fairly strong acidity at pH 1.0–2.0 (upper right). The pH of the intestinal lumen increases on average to 6.1 in the duodenum, 7.1 in the middle small intestine, and 7.5 in the distal small intestine (right middle). Thereafter, the pH temporarily reduces to approximately 6.0 near the cecum at the entrance of the large intestine and increases toward the rectum and becomes around 7.0 near the exit of the large intestine (lower right).

The pH gradient in the human body serves as the driving force of various vital reactions, such as intracellular adenosine triphosphate (ATP) synthesis and oxygen transfer in muscle tissues. In particular, because the gastrointestinal tract is constantly exposed to the external environment owing to the ingestion of foods, the gastrointestinal pH provides an optimal environment for the bactericidal effects of acids and the actions of digestive and metabolic enzymes. For instance, strong bactericidal action is required in the stomach considering that it experiences the most exposure to the external environment. Therefore, the gastric juice has a fairly strong acidity of pH 1.0–2.0 (**Figure 1**). To maintain this strong acidity, the stomach exhibits a regulatory function via gastrin that promotes hydrochloric acid secretion when pH increases to >3.0 (Daniels and Allum, 2005). This action appears to be a major factor that suppresses microbial colony formation in the stomach (Williams, 2001). Furthermore, it is known that the enzyme pepsin that is present in the gastric juice is activated at pH 1.0–2.0.

However, there are several bacteria that can survive in such highly acidic extreme environments. An example is *Helicobacter pylori*, which infects more than half of the world’s population (Santos et al., 2020) and is a clear risk factor for chronic gastritis, gastric ulcers, and stomach cancer (Camilo et al., 2017). The fact is that the bacteria prefer a rather neutral or near-neutral pH in the test tube and do not grow in a highly acidic environment such as that in the stomach, but rather may die. However, this bacterium has been shown to survive and grow in extreme environments by neutralizing gastric acid by acid-dependent activation of urease in the bacterium to produce ammonia (Scott et al., 1998). In addition, this bacterium is known to bind to and inhabit the gastric mucin layer, which maintains a moderate pH gradient. One study showed that all strains of *H. pylori* bind to relatively large mucins at low pH, regardless of host blood type, whereas only Le^b^-binding blood-group antigen-binding adhesin (BabA)-positive strains bind to Le^b^-positive mucins at neutral pH (Lindén et al., 2004). Thus, it is suggested that gastric pH has a strong influence on the binding and colonization of *H. pylori* to gastric mucins. Furthermore, gastric pH appears to influence the site of *H. pylori* infection. The usual site of *H. pylori* infection in the human stomach is not the fundus of the stomach, where acid secretion occurs, but the antrum of the stomach, indicating that the optimal pH for the growth of this bacterium is in the antrum of the stomach (Lee et al., 1995).

Although there are not many commensal bacteria that live in the stomach, some bacteria that cause intestinal infections survive the extreme environment of the stomach and make their way to the intestinal tract. A typical example is *Salmonella enterica*. *S. enterica* is a leading zoonotic pathogen and a major cause of mortality in people worldwide. After ingestion, *S. enterica* invades the intestinal epithelium of the ileum and colon, causing enteric salmonellosis characterized by fever, abdominal pain, vomiting, and diarrhea, or disseminates systemically to cause sepsis (Knodler and Elfenbein, 2019). This bacterium has a very specific characteristic, which is that the bacterium itself is not harmful to the human body when ingested orally, but it causes the severe symptoms described above when the bacterium is ingested in food. This is because *S. enterica* is killed by stomach acid when only the bacteria are ingested, but foods such as beef and egg whites temporarily raise the pH of the surrounding environment, allowing even acid-sensitive *S. enterica* to survive on the food surface (Waterman and Small, 1998). In this study, the authors inoculated ground beef with several pathogens and tested their ability to survive under acidic conditions of pH 2.5 at 37°C for 2 hours. The results showed that *Campylobacter jejuni* and *Vibrio cholerae*, which are acid-sensitive pathogens similar to *S. enterica*, also survived on the food surface, as did *S. enterica*, and repopulated when returned to optimal growth conditions. Other previous study showed that *Salmonella* spp. acquire an acid tolerance response (ATR), the ability to survive in extremely acidic conditions (pH 3) after one generation of culture at sublethal pH (pH 4.5-5.5) (Foster and Hall, 1990). Thus, although highly acidic stomach acid plays a very important role as the first line of defense against orally introduced pathogens, it does not seem to protect against all of them against food-borne pathogens.

## Intestinal pH and its roles

The pH of the intestinal tract of a healthy human has been investigated primarily by a pH-sensitive radiotelemetry capsule that passes freely through the digestive tract for up to 48 hours without the restrictions of normal ambulation (Evans et al., 1988), or by continuously measuring luminal pH in the small and right large intestine with an oral tube-mounted pH electrode (Nugent et al., 2001). First, the pH of the small intestinal tract, which is constantly exposed to the strong acids of gastric juice discussed in the previous chapter, was reviewed in detail in a recent meta-analysis (Abuhelwa et al., 2016). According to this report, the average pH value of the small intestinal tract is 6.1 in the duodenum, which is in direct contact with the stomach, rising to 7.1 in the middle part of the small intestine and reaching 7.5 in the distal part of the small intestine (**Figure 1**). The pH of the intestinal lumen increases to approximately 6.5 in the proximal small bowel and to approximately 7.5 near the ileum in the distal small bowel. Thereafter, it temporarily reduces to approximately 6.0 near the cecum at the entrance of the large intestine and then increases toward the rectum, where it reaches to around 7.0 near the exit of the large intestine (Crawford and Brooke, 1955; Pye et al., 1990; Nugent et al., 2001, **Figure 1**). The large intestine is responsible for numerous functions, such as the fermentation of carbohydrates and amino acids, absorption of minerals and bile acids, synthesis of vitamins, metabolism of various other substances, and excretion of unwanted substances (Cummings, 1997). In the proximal colon including the cecum, the fermentation of dietary fiber and saccharides by intestinal bacteria is active, and a large amount of SCFAs and their metabolites are produced (Nicholson et al., 2012; Baothman et al., 2016; Yamamura et al., 2020); therefore, it temporarily becomes acidic (approximately pH 6.0) in this area (**Figure 1**). In the distal colon, dietary fiber and saccharides are fermented by intestinal bacteria, and the SCFAs produced are rapidly absorbed from the intestinal wall by passive transport, so the closer to the rectum, the lower the amount of SCFAs. Furthermore, with the progress in amino acid fermentation, the proportion of alkaline metabolites increases, resulting in the pH becoming almost neutral (Conlon and Bird, 2015).

The increased production of SCFAs results in weakly acidic pH conditions in the intestinal tract, which play an important role in maintaining human health. For example, this condition is considered to exert the effect of reducing the solubility of toxic cholic acids, promoting the absorption of minerals and inhibiting the absorption of ammonia and other amines by producing ${\text{NH}}_{4}^{+}$ from NH_3_ *_via_* proton dissociation (Wong et al., 2006). Moreover, weakly acidic pH conditions in the intestinal tract have been shown to contribute significantly to the absorption of vitamins, electrolytes, and iron, as well as to the activation of digestive enzymes (Chernelch et al., 1970; Ohta et al., 1995; Fallingborg, 1999). It has also been reported that weakly acidic conditions in the intestinal tract strongly inhibit the growth of pathogenic *Clostridium*. More specifically, it was reported that germination and growth of *Clostridium difficile* from spores was greatly retarded when the medium was acidic (Kochan et al., 2018). As such, several studies have been conducted on the impact of pH on changes in the microbiome. In general, most opportunistic bacteria prefer to grow in near-neutral pH conditions (pH 6.0-7.0) and do not grow well in acidic conditions (pH ≤ 5.5). For example, some bacteria can grow at a wide range of pH values, such as *Bacteroides* (Duncan et al., 2009), while others are inhibited at acidic pH, such as *Veillonella* and *Streptococcus* (Bradshaw and Marsh, 1998). Furthermore, butyric acid bacteria such as *Faecalibacterium* and *Roseburia* are known to grow better at a weakly acidic pH (pH 5.5) than at a nearly neutral pH (pH 6.7) and produce more butyric acid (Walker et al., 2005). This stimulation of butyric acid production is considered to nourish colon cells and protect them from hydrogen peroxide-induced DNA damage (Rosignoli et al., 2001). Furthermore, weakly acidic pH (pH 5.5) may slightly inhibit propionate production as a result of restricted growth of propionate-producing species such as Bacteroides (Walker et al., 2005). Propionate is the second preferred energy source for colon cells after butyrate (Clausen and Mortensen, 1994), has anti-inflammatory properties, and can play an important role in the treatment of inflammatory bowel disease (Tedelind et al., 2007). Other studies have shown that a decrease in colonic pH prevents the growth of pathogenic bacteria, especially *Enterobacteraciae*, which are generally sensitive to low pH (Roe et al., 1998; Hirshfield et al., 2003). Interesting results have also been shown in *in vitro* experiments in which pH values of 33 representative human E. coli species were measured at three levels of approximately 5.5, 6.2, and 6.7 (Duncan et al., 2009). In this study, they found that the eight representative *Bacteroides* spp. tested grew little at pH 5.5, as did *E. coli*. They also reported that *Bacteroides* spp. accounted for 27% of the 16S rRNA sequences detected at pH 5.5, but 86% at pH 6.7, and conversely, butyrate-producing Gram-positive bacteria accounted for 50% of all 16S rRNA sequences at pH 5.5, but none at pH 6.7. From this study, the authors concluded that a major group of Gram-negative bacteria is inhibited at a weakly acidic pH, creating a niche available to lower pH-resistant microorganisms. In addition, another *in vitro* experiment using indigestible polysaccharides as growth substrates showed that a one-unit pH shift causes significant changes in the composition of the human colonic microbiota, particularly with Gram-negative Bacteroides species becoming dominant at pH 6.5 and Gram-positive Firmicutes increasing at pH 5.5 (Walker et al., 2005).

Thus, it is clear that pH can promote or inhibit the growth of certain bacteria. other effects of pH on the microbiome include the promotion or inhibition of fermentation by the microbiome. In one *in vitro* study, in a pH-controlled fermenter, more butyrate was produced than acetic acid at pH 5.5, but the opposite was true at pH 6.5 (Walker et al., 2008). Another similar study reported that *in vitro* cultures with mixed fecal microflora produced lactic acid in the pH range of 5.2 to 6.4, with the highest production under mildly acidic conditions (pH 5.9) (Belenguer et al., 2007). They also reported that at low pH (<5.2), lactic acid production increased slightly, but lactic acid utilization was strongly inhibited and lactic acid accumulated.

In addition, a recent study showed that *V. cholerae*, which is transmitted by oral infection from contaminated food and water and has a very high disease burden in developing countries, has an increased swimming speed using flagellar motility at alkaline pH, which results in enhanced intestinal mucus invasion (Nhu et al., 2021). Thus, the production of SCFAs by intestinal bacteria and the resulting weakly acidic conditions in the intestinal tract have a variety of health effects, such as promoting the elimination of toxic substances from the intestinal tract, promoting the absorption of nutrients, and inhibiting the growth of pathogenic bacteria.

## Clinical significance of fecal pH

**Figure 2:**
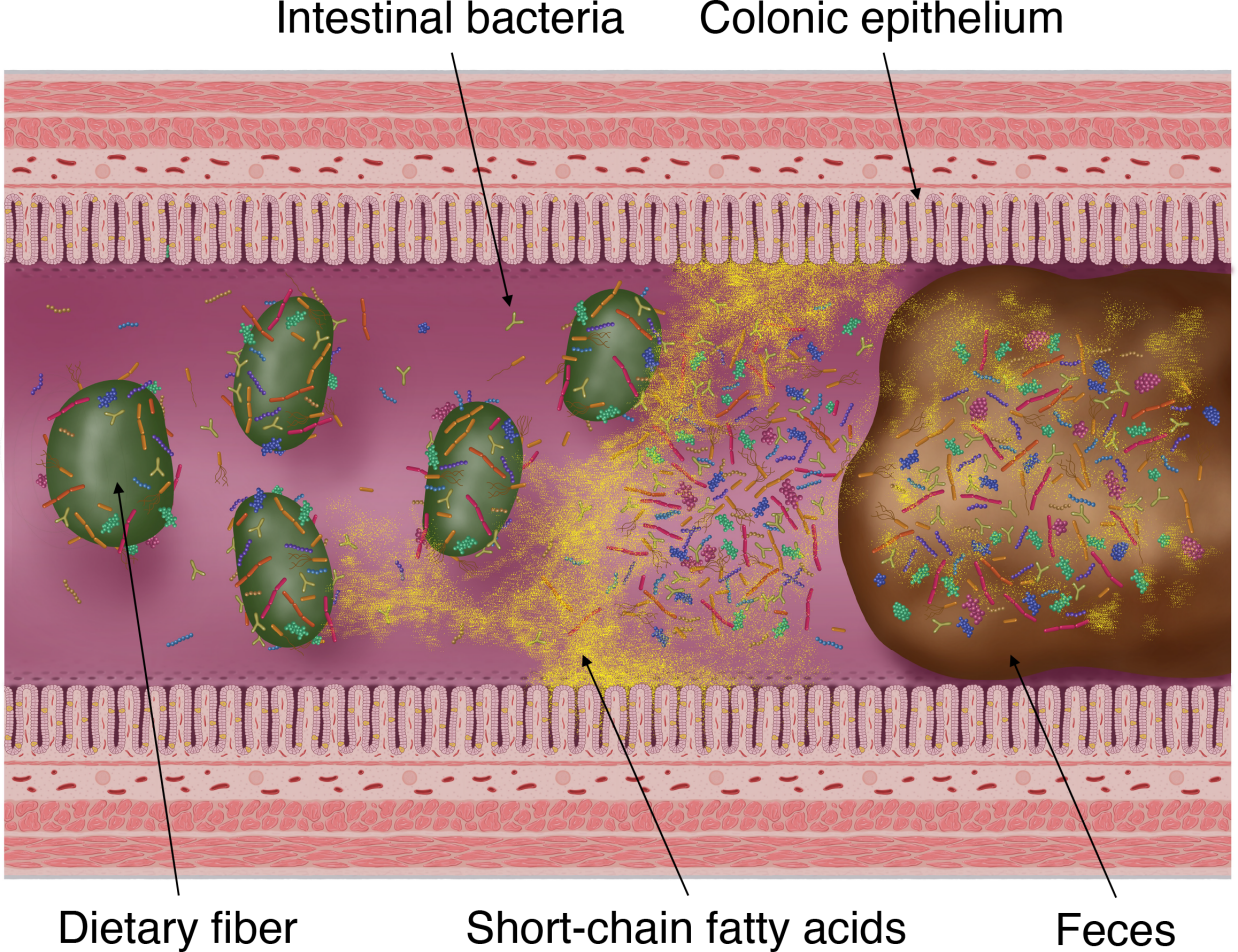
Fermentation of dietary fiber by intestinal bacteria, production of short-chain fatty acids (SCFAs) and its absorption into the colonic epithelium and excretion into faces. SCFAs are the products of gut microbial fermentation of mainly host-derived dietary fibers. Approximately 95% of SCFAs produced by the gut microbiota are absorbed by the colonic epithelium *via* rapid passive transport, whereas the remaining 5% are excreted in the faces.

SCFAs produced by intestinal bacteria, mainly by fermenting carbohydrates (dietary fiber and saccharides), are rapidly absorbed from colonic epithelial cells into the host body, stimulating the absorption of water and sodium in the intestinal tract (**Figure 2**) (Fleming and Arce, 1986). Moreover, this fermentation of carbohydrates into SCFAs is known to reduce the osmotic pressure in the intestinal lumen and either prevent or alleviate osmotic diarrhea (Jiang and Savaiano, 1997). Conversely, the malabsorption of carbohydrates and SCFAs into the intestinal tract increases the SCFA concentration excreted in feces and simultaneously elevates fecal osmotic pressure. This causes the water in the intestinal tract to transfer to feces, resulting in osmotic diarrhea (Blattner, 1961; Hammer et al., 1989; Hammer et al., 1990).

Fecal pH is currently used in clinical practice as an indicator of carbohydrate malabsorption in the colon and osmotic diarrhea (Maffei et al., 1984; Fine and Schiller, 1999; Podolsky et al., 2015; Karu et al., 2018). It should be noted, however, that there are quite a number of factors that affect intestinal and fecal pH, as discussed in the next chapter, and therefore their significance in clinical practice is still very limited.

## Factors affecting intestinal and fecal pH

**Figure 3:**
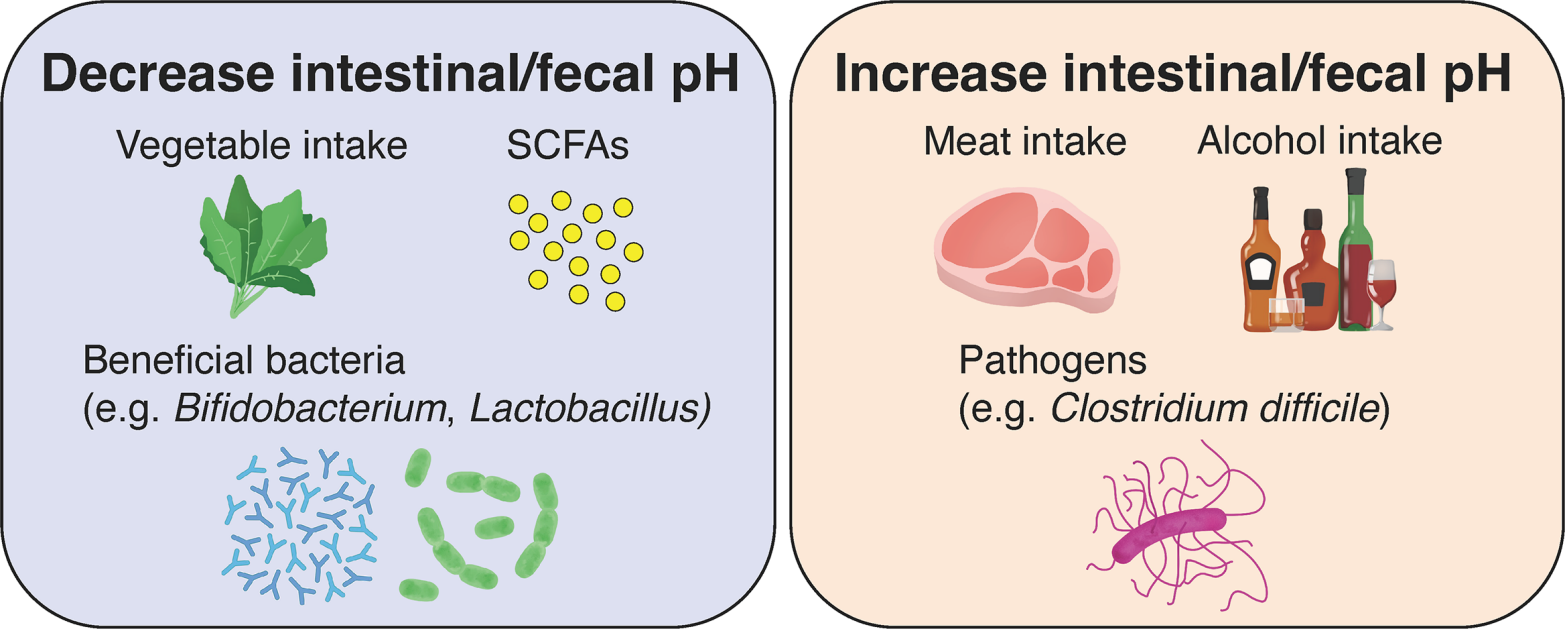
Effects of diet, gut microbiota, and their metabolites on intestinal and fecal pH in healthy individuals. The consumption of vegetables and low-fat diets, the production of short-chain fatty acids (SCFAs) by gut microbiota and the growth of beneficial bacteria lead to a decrease in intestinal and fecal pH. Conversely, the consumption of meat and high-fat diets, alcohol, and the growth of pathogenic bacteria lead to an increase in intestinal and fecal pH.

As described above, fluctuations in intestinal pH caused by metabolites of intestinal bacteria play an extremely important role in maintaining the functions and health of the large intestine. Meanwhile, fluctuations in the gut microbiota affect the digestion of food ingested by us, the host, and the increase or decrease in metabolites, which in turn cause fluctuations in intestinal and fecal pH (**Figure 3**). In the present review, we examine the factors affecting intestinal microbiota and summarize its relationship with intestinal and fecal pH, which can be noninvasively and relatively easily measured.

### Age

**Figure 4:**
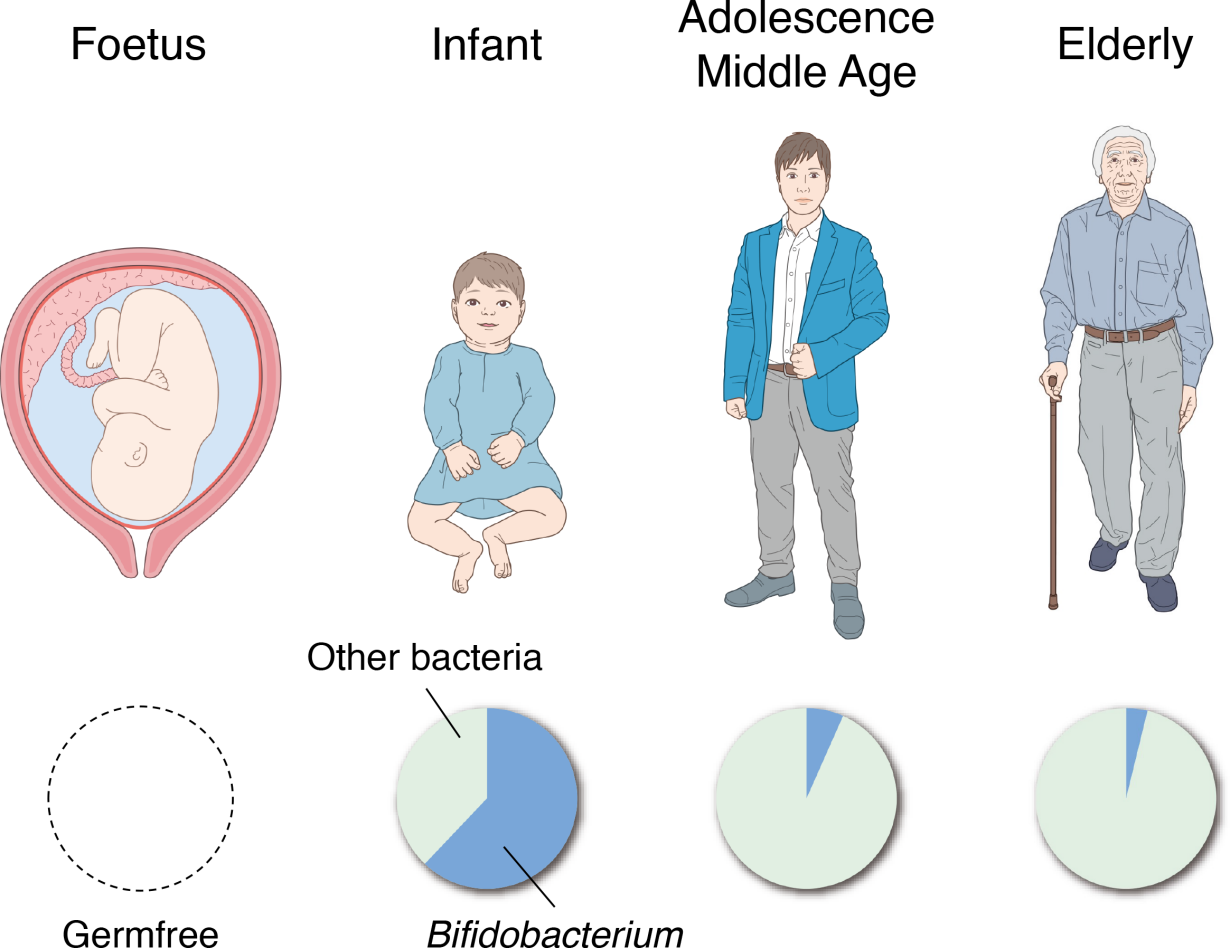
Changes in the gut microbiota with age. The intestines of human fetuses are completely sterile (left). The genus *Bifidobacterium* becomes the predominant bacterial group in the gut in the first 2 weeks of life (second from the left), and the percentage dramatically decreases through adolescence and middle age (second from the right). In the faces of the elderly, the proportion of *Bifidobacterium* is further reduced and the presence of putrefactive bacteria is relatively increased (right).

The intestines of human fetuses are known to be completely sterile. Exposure to maternal and environmental bacteria at birth is considered to initiate the formation of the gut microbiota (**Figure 4**) (Arrieta et al., 2014). Typically, the first bacteria to settle in the intestines are facultative anaerobes such as *Enterococcus*, *Staphylococcus* and *Enterobacteriaceae* (Tsuji et al., 2012). Thereafter, obligate anaerobes such as *Bifidobacterium*, *Bacteroides*, and *Clostridium* appear and *Bifidobacterium* becomes the predominant bacterial group in the intestines approximately 2 weeks after birth (**Figure 4**) (Mitsuoka, 2014). These changes in the gut microbiota during infancy greatly affect the changes in fecal pH. The fecal pH of infants at 1–2 h after birth is approximately 6.0 and reduces to around pH 5.0 with the start of breastfeeding (Grütte and Haenel, 1980). The fecal pH of breastfed infants becomes acidic because the count of *Bifidobacterium*, which metabolizes human milk oligosaccharides in breast milk to acidic final products centered on lactic and acetic acids, increases in the intestines (Stiles, 1996); fecal pH becomes increasingly alkaline with the consumption of a diet similar to that of adults as the abundance of *Bifidobacterium* relatively decreases (Haenel et al., 1970; Kurokawa et al., 2007). However, in recent years, numerous babies, particularly in developed countries, have reportedly been born with no or significantly less *Bifidobacterium* than in the past or in developing countries (Grześkowiak et al., 2012; Henrick et al., 2018). These babies exhibited a markedly higher fecal pH than babies with the normal *Bifidobacterium* population. They are known to have larger amounts of pathogens and mucus-eroding bacteria among intestinal microbiota and have signs of chronic enteric inflammation (Duar et al., 2020a; Duar et al., 2020b). These results suggest that the existence of *Bifidobacterium* and a low intestinal pH are important for protecting the intestinal environment of infants.

In the feces of the elderly, the abundance of *Bifidobacterium* decreases, and the abundance of putrefactive bacteria such as *Escherichia coli* and *Clostridium perfringens* relatively increases (Mitsuoka, 1990), thereby resulting in higher fecal pH. A study that classified 66 healthy individuals (aged 21–82 years) into groups as those aged >40 years and those aged<40 years showed no significant difference in fecal pH between both groups (Pye et al., 1990); therefore, it is suggested that fecal pH does not change significantly in adolescence, early middle age, and late middle age. However, these studies only observed the association between specific bacterial species and fecal pH in a cross-sectional manner, and further studies are needed to elucidate the causal relationships and mechanisms of how these bacterial species affect the intestinal tract or fecal pH with age, and how they affect the onset and progression of disease.

### Diet

Activities involving food in the large intestine include SCFA production via the fermentation of carbohydrates (particularly dietary fiber) and proteins by intestinal bacteria, production of amines and fatty acids with branched alkyl groups by amino acid metabolism, and bile acid metabolism and absorption used in lipid digestion. Therefore, the diet and nutrients we consume appear to greatly impact fecal pH via the production, metabolism, and absorption of fatty acids and bile acids in the large intestine. This section focuses on the effects of diet and nutrients on fecal pH and summarizes the findings achieved to date.

An interventional study in which women with low iron (*n* = 32) were orally administered inulin or placebo 3 times/day (approximately 20 g/day) for 4 weeks found that inulin intake significantly decreased stool pH (Petry et al., 2012). Moreover, the same result has been reported in an animal experiment using rats (Ohta et al., 1995). Furthermore, an interventional study investigated the changes in fecal pH in a group that consumed a high-protein and high-carbohydrate diet and another group that consumed a high-protein and low-carbohydrate diet; it was observed that fecal pH became increasingly acidic in the former and increasingly alkaline in the latter. The interventional study also examined intestinal microbiota whose abundance ratio actually changed and found that the *Roseburia* spp. and *Eubacterium rectale* of the *Clostridium coccoides* group decreased in the group fed with a high-protein and low-carbohydrate diet (Russell et al., 2011). Furthermore, in an experiment where the initial pH condition in the culture of symbiotic bacteria was changed, when compared at pH 5.5 and pH 6.5, it was observed that the growth of Bacteroidetes was inhibited at pH 5.5, whereas the proliferation of *Roseburia* spp. and *E. rectale* of the *C. coccoides* group was particularly promoted (Duncan et al., 2009). These results suggested that the weakly acidic condition caused by the fermentation of carbohydrates affects the compositional change of the gut microbiota.

Conversely, in an intervention study where 1.4 g or 2.8 g of xylooligosaccharide was orally administered to 32 healthy adults for up to 8 weeks, there were no effects on fecal pH (Li et al., 2014). Furthermore, a recent blinded, randomized, crossover dietary intervention study included 20 healthy young adults (18-45 years of age) who were fed high and low SCFA diets for 21 days with a 21-day washout in between (Gill et al., 2022). However, there was no significant difference in fecal pH between the high and low SCFA diet groups. In addition, a single-blind randomized controlled trial of 52 patients with quiescent Crohn’s disease or ulcerative colitis conducted in the UK in 2020 showed that dietary intervention did not alter fecal pH (Cox et al., 2020). In this study, patients were randomly assigned to a low-fermentable oligosaccharide, disaccharide, monosaccharide, polyol (FODMAP) diet group and a control diet group with dietary advice, and after 4 weeks of intervention, fresh stool pH was measured. Relatedly, a 2022 meta-analysis of nine randomized controlled trials of low FODMAP diets in patients with sensitive bowel syndrome similarly reported no effect of dietary intervention on fecal pH (So et al., 2022). The authors also concluded in this study that the effect of a low FODMAP diet on the colonic microbiome in IBS patients is specific to *Bifidobacterium*, with no consistent effects on other microbiome indicators such as microbiota diversity or stool SCFA concentrations. Thus, discrepancies between highly blinded and randomized studies of dietary interventions in similar subjects still suggest that fecal pH may not be consistently linked to any particular diet or nutrient.

A study on infants reported that the fecal pH of breastfed infants was significantly lower than that of formula-fed infants (Langhendries et al., 1995). One of the reasons for this difference is that *Bifidobacterium* is the most predominant bacterial group in breastfed 1-month-old infants, and a large amount of SCFAs is produced (see “Age” section). However, reportedly, the fecal pH of breastfed infants with almost no *Bifidobacterium* was lower than that of formula-fed infants; thus, the reason remains unclear (Willis et al., 1973).

### Drug intake

Drugs are also metabolized in the large intestine. However, drugs such as laxatives, antidiarrheals, and antibiotics are mainly directly associated with changes in intestinal microbiota and pH. It has been reported that taking laxatives lowers fecal pH, whereas taking antidiarrheals increases fecal pH (Lewis and Heaton, 1997). In addition, a study investigating the fecal pH of patients with colorectal cancer (CRC) reported that these patients with alkaline fecal pH are less prone to diarrhea (Holma et al., 2013), and this was associated with the tendency of feces to be excreted when pH decreases.

Regarding antibiotics, despite differences depending on the type of drugs used, they are known to typically kill major SCFA-producing bacteria and increase fecal pH (Osuka et al., 2012). Therefore, studies focusing on the relationship between fecal pH and diseases that will be described later are mostly attentive to the presence or absence of antibiotic administration. Furthermore, acarbose, an antidiabetic drug that is a type of α-glucosidase inhibitor that delays carbohydrate digestion and intestinal absorption, has been reported to significantly increase total SCFA, acetic acid, and butyric acid concentrations in feces, significantly lowering fecal pH (Holt et al., 1996). If future interventional studies can clarify the causal relationship by longitudinal and long-term monitoring of changes in intestinal and fecal pH before and after drug administration, along with changes in the gut microbiota and its metabolites, intestinal and fecal pH may be utilized as indicators of intestinal environmental dysbiosis. However, even in such a case, it is necessary to keep in mind that the clinical application of pH should take into account confounding factors such as pH measurement method, diet, disease, and other concomitant medications.

### Diseases

In recent years, a number of diseases in which the composition of intestinal bacteria and their metabolites are deeply involved have been revealed. This section summarizes the relationship between diseases and intestinal microbiota as well as fecal and intestinal pH (**Table 1**).

**Table 1:** Relationship between diseases and intestinal microbiota as well as fecal and intestinal pH.

Disease	Fecal pH	Colonic lumen pH	Fecal SCFA levels	Microbiota	Ref.
Colorectal Cancer	Alkalinity	–	–	–	Pye et al., 1990
	Unchanged	–	–	–	Hove et al., 1993; Holma et al., 2012
	Alkalinity	–	Lower (Total organic acid, acetic acid, butyric acid, propionic acid, valeric acid)	Total bacteria counts ↓	Ohigashi et al., 2013
				*Clostridium coccoides* group ↓	
				*Clostridium leptum* ↓	
				*Bacteroides fragilis* group ↓	
				*Bifidobacterium* ↓	
				*Atopobium* cluster ↓	
				Enterobacteriaceae ↓	
				*Staphylococcus* ↓	
Colonic Polyps	Acidity	–	–	–	De Kok et al., 1999
	Alkalinity	–	Lower	Total bacteria counts ↑	Ohigashi et al., 2013
				*Clostridium leptum* ↓	
				*Bacteroides fragilis* ↓	
				Staphylococcus ↓	
Pseudomembranous Colitis	Alkalinity	–	–	–	Gupta et al., 2016
Ulcerative Colitis	Acidity	–	Lower	–	Vernia et al., 1988a
	Acidity	–	Lower (Lowest in severe colitis and pancolitis)	–	Vernia et al., 1988b
	–	Acidity	–	–	Macfarlane et al., 1992
Crohn’s disease	Acidity	–	Unchanged	–	Vernia et al., 1988a
Systemic inflammatory response syndrome	Alkalinity	–	Lower (Total organic acid, acetic acid, butyric acid, propionic acid)	*Bifidobacterium* ↓	Shimizu et al., 2006
				*Lactobacillus* ↓	
				*Veillonella* ↓	
				Enterobacteriaceae ↓	
				*Staphylococcus* ↑	
				*Pseudomonas* ↑	
Type 2 diabetes mellitus	Unchanged	–	Lower	–	Sato et al., 2014
Anorexia nervosa	Alkalinity	–	–	–	Morita et al., 2015

-, not tested; ↓, decreased; ↑, increased.

#### Colorectal cancer and colonic polyps

Until around 1990, the feces of patients with CRC were reported to be alkaline (Pye et al., 1990). However, since 1990, some reports have stated that fecal pH does not change even with CRC (Hove et al., 1993; Holma et al., 2012). Moreover, a study on colon polyps reported that fecal pH is conversely acidic in the group diagnosed with >1 villous colorectal (renal tubular) adenoma exceeding 1 cm in diameter, with a high risk of having moderate or severe dysplasia (De Kok et al., 1999). One relatively new report on CRC and fecal pH involved a study of 93 patients and 49 healthy volunteers; in this report, patients with CRC exhibited a higher fecal pH and patients with colonic polyps exhibited a fecal pH between that of healthy individuals and that of patients with CRC (Ohigashi et al., 2013). Therefore, the specific relationship between CRC and fecal pH remains undetermined, and larger-scale epidemiological studies are required in the future.

Regarding the relationship between the gut microbiota and CRC, findings of the possible involvement of *Fusobacterium* in the development and progress of CRC have consecutively been reported in recent years (Kostic et al., 2012; Kostic et al., 2013; Tahara et al., 2014). Conversely, in patients with CRC, the proportion of obligate anaerobes such as the *Clostridium coccoides* group, *Clostridium leptum* subgroup, *Bacteroides fragilis* group, and *Bifidobacterium* and *Atopobium* cluster was typically low; moreover, the proportion of *Enterobacteriaceae* and *Staphylococcus* was low (Kostic et al., 2012; Kostic et al., 2013; Tahara et al., 2014). Many of these bacteria are typical SCFA-producing bacteria and may be associated with the changes in the fecal pH (particularly changes to alkalinity) of patients with CRC.

#### Pseudomembranous colitis

For confirming the diagnosis of pseudomembranous colitis, a combination of the toxin and antigen tests has been shown to be effective. This is because when only either one was positive, there were several misdiagnoses. The fecal pH of patients with pseudomembranous colitis was reportedly alkaline in both positive groups (Gupta et al., 2016). Furthermore, the study reported that there was no significant difference in fecal pH of either-positive or both-negative patients. This type of colitis is one of the infectious colitis caused by the abnormal proliferation of *Clostridium difficile*, a spore-producing obligate anaerobe (Moreno et al., 2013). It has been found that *C. difficile* rarely inhabits feces with a pH below 6.0 (Miyazaki et al., 1992), and it may be associated with the alkaline fecal pH of patients with pseudomembranous colitis.

#### Ulcerative colitis and Crohn’s disease

It has been clarified that fecal pH tends to be lower in patients with UC and Crohn’s disease (CD) compared with that in healthy individuals (healthy individuals: pH 7.0, UC: pH 6.6, and CD: pH 6.8) (Vernia et al., 1988b). In particular, in patients with UC, it has been reported that fecal pH decreases with the increase in severity from the remission phase, mild and moderate to severe (remission phase: pH 7.2, mild: pH 6.4, moderate: pH 6.3, and severe: pH 6.2) (Vernia et al., 1988a). Remarkably, fecal SCFA levels were significantly lower in patients with UC than in healthy individuals (Treem et al., 1994; Nemoto et al., 2012; Zhuang et al., 2019), and this may be related to the increased fecal pH in these patients. Similar to the fecal results, colonic lumen pH has been shown to decrease in patients with active UC (Macfarlane et al., 1992). Furthermore, an interventional study where exclusive enteral nutrition (EEN) was administered to pediatric patients with CD reported a gradual increase in fecal pH and an alleviation of CD symptoms on days 15, 30 and 60 after the initiation of EEN (Gerasimidis et al., 2014).

With regard to intestinal bacteria, the phylum Proteobacteria, adherent-invasive *E. coli*, *Fusobacterium*, and *Ruminococcus gnavus* are known to adversely affect the pathogenesis of UC and CD. Moreover, *Bifidobacterium*, Groups IV & XIVa *Clostridium*, *Facalibacterium prausnitzii*, *Roseburia* spp., *Suterella* spp., *Bacteroides*, and *Saccharomyces cerevisiae* reportedly decrease in patients with inflammatory bowel disease (Sartor and Wu, 2017). Among them, the increase in *Fusobacterium* and decrease in *Bacteroides*, *Bifidobacterium*, and *Clostridium* are the same as those observed in patients with CRC (see “Colorectal Cancer and Colonic Polyps” section) and may be associated with the changes in fecal pH.

#### Other diseases

In addition to the colonic diseases mentioned above, various other illnesses have been found to be associated with fecal pH. There is a study about systemic inflammatory response syndrome (SIRS), a general term for serious conditions such as sepsis caused by burns or accidents, investigating the relationship between symptoms, the gut microbiota, fecal SCFA levels and fecal pH of patients transported by ambulance and treated in the intensive care unit (ICU). In this study, the fecal pH of patients with SIRS was more alkaline than that of healthy individuals, which may be attributed to the fact that these patients exhibited less number of *Bifidobacterium* and *Lactobacillus* and lower fecal SCFA levels compared with healthy individuals. Similarly, more elevated fecal pH has been reported in patients with severe SIRS (Shimizu et al., 2006), and abnormal fecal pH (below 6.0 and above 7.2) in patients with severe SIRS showed a significant positive correlation with an increased risk of death and bacteremia (Osuka et al., 2012). These findings may be attributed to the destruction of the gut microbiota by H_2_ blockers and antibiotics that are administered to severe ICU patients, thereby resulting in a decrease in the amount of SCFAs produced, leading to the feces becoming extremely alkaline, or resulting in the damage to the intestinal epithelium by harmful bacteria that causes lactose malabsorption, leading to the feces becoming extremely acidic. Furthermore, it has been reported that the supplementation of a decreased amount of *Bifidobacterium* and *Lactobacillus* with the symbiotic therapy (a combination of probiotics and prebiotics) for patients with SIRS increases fecal SCFA levels, lowers pH and reduces the incidence of enteritis, pneumonia and bacteremia (Shimizu et al., 2009). The fecal pH of patients with SIRS may be higher than that of healthy individuals owing to antibiotic administration. However, the fact that symbiotic therapy resulted in decreased incidence of various infections along with increased fecal SCFA levels and decreased fecal pH suggests that fecal pH is important for understanding the systemic immune state.

Other diseases that reportedly alter fecal pH and fecal SCFA levels in addition to SIRS include type 2 diabetes (T2DM) and anorexia nervosa. Two studies have reported that fecal pH remained unchanged, and only the total fecal SCFA levels were reduced in patients with T2DM (Sato et al., 2014), whereas the feces became alkaline in patients with anorexia nervosa (Morita et al., 2015). The reason for alkaline feces in the case of anorexia may be because food is not orally consumed. Combined with the description in the Diet section, carbohydrate intake may be the key to the acidification of fecal pH in any case.

Several other diseases have been reported to be associated with the gut microbiota, such as obesity (Ley et al., 2006; Turnbaugh et al., 2006), metabolic syndrome (Le Chatelier et al., 2013; Hartstra et al., 2015), irritable bowel syndrome (Ford et al., 2009), liver diseases (alcohol-related liver disease (Yan et al., 2011), nonalcoholic fatty liver disease (Harte et al., 2010), liver cirrhosis (Qin et al., 2014), and primary sclerosing cholangitis (Sabino et al., 2016)), arteriosclerosis (Kasahara et al., 2017), multiple sclerosis, (Jangi et al., 2016), and autism spectrum disorder (Finegold et al., 2002). However, no study has been conducted on the relationship between any of them and fecal pH. Further research is anticipated in the future.

### Other factors

In addition to the above-mentioned factors, there are other factors strongly associated with fecal and intestinal pH. In particular, fecal pH reportedly has a strong negative correlation with the abundance of *Bifidobacterium*, and in recent years, fecal pH has been proposed to be used as an indirect measurement index of the abundance of *Bifidobacterium* (Frese et al., 2017; Henrick et al., 2018). For example, the fecal pH of infants had continued to increase for approximately 100 years (from 1926 to 2017), suggesting that it is related to the decrease in the abundance of *Bifidobacterium* in infants in developed countries (Henrick et al., 2018). In the future, it may become possible to easily estimate the composition of intestinal bacteria by combining fecal pH and fecal metabolites. In addition, it has been demonstrated that intestinal pH is strongly affected by the fermentation of carbohydrates by intestinal microbiota and absorption of SCFAs from colonic epithelial cells as well as whole-gut transit time and fecal water content (Nugent et al., 2001; Karu et al., 2018). The technique of noninvasively analyzing and examining the intestinal environment by measuring fecal pH has already been applied in clinical examinations (see “Clinical Significance of Fecal pH” section). However, as noted above, intestinal and fecal pH are strongly influenced by a variety of factors, and their clinical significance and interpretation require considerable attention.

## Limitations

As discussed in this paper, intestinal and fecal pH appear to fluctuate with some correlation to disease status. However, it is also affected by a variety of other factors, such as aging, diet, and medications, and even within individuals, intra- and inter-day variations are expected to be very large. Regarding diet, various intervention studies have also been conducted, but the results on the effects of diet on intestinal and fecal pH have been inconsistent. Furthermore, differences in intestinal and fecal pH between diseases have not yet been studied, and it would be difficult to utilize them as markers to distinguish between diseases. Moreover, although we focused on the correlation of intestinal and fecal pH variation with SCFA in this review, the extent to which SCFA influences intestinal and fecal pH variation is not yet known. Furthermore, no quantitative and high-quality studies have yet been conducted to determine to what extent the variation in intestinal and fecal pH conversely affects the acidity of SCFA. In view of these facts, this research field in the future will first require standardization of intestinal and fecal pH measurement methods (measuring instruments, time of day, and methods of recording meals and medications up to the day before the day of measurement). Then, if it can be clarified to what extent intestinal and fecal pH fluctuates in conjunction with gut microbiota and intestinal metabolites such as SCFA, and in what degree of causal relationship, the possibility of utilizing intestinal and fecal pH as a simple tool for understanding the intestinal environment of each individual will be opened up.

## Conclusions

On the basis of the information summarized in this review, it can be perceived that fecal pH changes with a certain degree of clear cause-to-effect correlation depending on the individual’s condition and lifestyle, such as age, diet, disease conditions, drugs, the gut microbiota and its metabolites. However, it is also important to consider that fecal pH can change significantly during storage of fecal samples and that many factors can affect its value at the same time. Further limitations should be noted that the methods for evaluating intestinal and fecal pH have not yet been standardized, fresh samples are difficult to obtain for feces, and the intestinal pH is difficult to implement. Therefore, at present, the significance of intestinal and fecal pH values in human health is limited, and it is difficult to set a normal or abnormal threshold for fecal pH value that is universal to all people, and rather it is effective to observe changes that occur in the daily life of individuals. In this respect, clinical applications of fecal pH are promising, for example, for diseases such as inflammatory bowel disease, for which maintenance of remission is important, monitoring fecal pH in individuals over time may enable noninvasive and rapid understanding of pathological conditions. Furthermore, combined with indicators such as gut microbiota and metabolite concentrations in feces and blood, this technique will pave the way for the development of evidence-based biomarkers for various health issues as well as for digestive health.

## Author contributions

Conceptualization: RY, KI, KN, SY. Methodology: RY, KI, SY. Investigation: RY, KI, SY. Visualization: RY, KI, SY. Funding acquisition: RY, KI, KN, SY. Project administration: RY, KI, KN, SY. Supervision: KN, SY. Writing – original draft: RY, KI, SY. Writing – review & editing: RY, KI, KN, SY. All authors contributed to the article and approved the submitted version.
